# Modifications of the GH Axis Reveal Unique Sexually Dimorphic Liver Signatures for *Lcn13*, *Asns*, *Hamp2*, *Hao2*, *and Pgc1a*

**DOI:** 10.1210/jendso/bvae015

**Published:** 2024-01-31

**Authors:** Belen Brie, Andre Sarmento-Cabral, Florencia Pascual, Jose Cordoba-Chacon, Rhonda Denise Kineman, Damasia Becu-Villalobos

**Affiliations:** Instituto de Biología y Medicina Experimental (IBYME), Consejo Nacional de Investigaciones Científicas y Técnicas (CONICET), 1428 Ciudad de Buenos Aires, Argentina; Department of Medicine, Division of Endocrinology, Diabetes and Metabolism, University of Illinois at Chicago, Chicago, IL 60612, USA; Instituto de Biología y Medicina Experimental (IBYME), Consejo Nacional de Investigaciones Científicas y Técnicas (CONICET), 1428 Ciudad de Buenos Aires, Argentina; Department of Medicine, Division of Endocrinology, Diabetes and Metabolism, University of Illinois at Chicago, Chicago, IL 60612, USA; Department of Medicine, Division of Endocrinology, Diabetes and Metabolism, University of Illinois at Chicago, Chicago, IL 60612, USA; Research and Development Division, Jesse Brown Veterans Affairs Medical Center, Chicago, IL 60612, USA; Instituto de Biología y Medicina Experimental (IBYME), Consejo Nacional de Investigaciones Científicas y Técnicas (CONICET), 1428 Ciudad de Buenos Aires, Argentina

**Keywords:** growth hormone, STAT5b, cytochromes, liver, sexual dimorphism

## Abstract

Growth hormone (GH) modifies liver gene transcription in a sexually dimorphic manner to meet liver metabolic demands related to sex; thus, GH dysregulation leads to sex-biased hepatic disease. We dissected the steps of the GH regulatory cascade modifying GH-dependent genes involved in metabolism, focusing on the male-predominant genes *Lcn13*, *Asns*, *and Cyp7b1*, and the female-predominant genes *Hao2*, *Pgc1a*, *Hamp2*, *Cyp2a4*, *and Cyp2b9*. We explored mRNA expression in 2 settings: (i) intact liver GH receptor (GHR) but altered GH and insulin-like growth factor 1 (IGF1) levels (NeuroDrd2KO, HiGH, aHepIGF1kd, and STAT5bCA mouse lines); and (ii) liver loss of GHR, with or without STAT5b reconstitution (aHepGHRkd, and aHepGHRkd + STAT5bCA). *Lcn13* was downregulated in males in most models, while *Asns* and *Cyp7b1* were decreased in males by low GH levels or action, or constant GH levels, but unexpectedly upregulated in both sexes by the loss of liver *Igf1* or constitutive *Stat5b* expression. *Hao*, *Cyp2a4*, and *Cyp2b9* were generally decreased in female mice with low GH levels or action (NeuroDrd2KO and/or aHepGHRkd mice) and increased in HiGH females, while in contrast, *Pgc1a* was increased in female NeuroDrd2KO but decreased in STAT5bCA and aHepIGF1kd females. Bioinformatic analysis of RNAseq from aHepGHRkd livers stressed the greater impact of GHR loss on wide gene expression in males and highlighted that GH modifies almost completely different gene signatures in each sex. Concordantly, we show that altering different steps of the GH cascade in the liver modified liver expression of *Lcn13*, *Asns*, *Cyp7b1*, *Hao2*, *Hamp2*, *Pgc1a*, *Cyp2a4*, *and Cyp2b9* in a sex- and gene-specific manner.

Growth hormone (GH), a key hormone regulating postnatal growth, plays a major role in metabolic function after puberty [1], and GH deficiency is associated with increased adiposity, accumulation of fat in the liver, and the development of nonalcoholic fatty liver disease (NAFLD) and nonalcoholic steatohepatitis (NASH), which is reduced by GH replacement [2]. GH signals through the GHR/JAK2/STAT5b (growth hormone receptor/Janus kinase 2/signal transducers and activators of transcription 5b) pathway to maintain hepatic expression of *Igf1* [3], and secreted insulin-like growth factor 1 (IGF1) exerts a negative feedback on pituitary GH. This pathway also regulates the expression of a large subset of hepatic metabolic genes, many in a sex-dependent fashion [4]. Sex bias in liver gene transcription may be envisioned as an orchestrated action by which GH regulates key genes according to their function, to accommodate liver metabolic demands in relation to sex [5, 6].

In rodents, GH secretion is sexually fine-tuned, with high intermittent pulses in males, and a more constant release pattern in females. Sexually dimorphic GH secretion is also observed in humans, although to a lesser extent [7]. It has been established that the male-dependent pattern of GH secretion leads to intermittent activation/phosphorylation of hepatic STAT5b and subsequent binding to DNA, while the female GH release pattern leads to continuous pSTAT5b and DNA binding [8, 9]. In this respect, persistent hepatocyte pSTAT5b (female pattern) repressed more than 90% of hepatic male-biased genes, and de-repressed 60% of hepatic female-biased genes [10].

Of clinical importance, there is a differential susceptibility between sexes to liver disease. Specifically, steatosis, nonalcoholic steatohepatitis, and progression to hepatocellular carcinoma (HCC) predominate in men, while primary biliary cirrhosis, autoimmune hepatitis, or alcoholic liver disease predominate in women [11-14]. Given the sexual dimorphism of GH-induced hepatic gene expression, it is logical to assume that the sex-dependent predisposition to liver diseases may be in part regulated by GH/GHR/Jak2/Stat5b-signaling mechanisms. In this context, we focused on dissecting the steps of the GH regulatory cascade that influence liver gene sexual dimorphism. We centered our analysis on studying genes that play key roles in liver metabolism and disease, and therefore may be relevant in the sexual dimorphism for the susceptibility to liver disease. Furthermore, the dependence of their expression on the different steps of GH signaling in each sex has not been studied. Two GH-dependent male-predominant genes were studied: lipocalin 13 (*Lcn13*) and asparagine synthetase (*Asns*), and 3 female-predominant genes: hydroxyacid oxidase 2 *(Hao2*), peroxisome proliferator-activated receptor-gamma coactivator 1 alpha (*Pgc1a*), and hepcidin antimicrobial peptide 2 *(Hamp2*).

Hepatic LCN13 (also known as odorant binding protein2a, OBP2a) has a striking sexual dimorphism and marked GH dependence [15]. In male mice, it suppresses gluconeogenesis and lipogenesis, increases fatty acid β oxidation, and enhances insulin sensitivity in adipocytes [16-18]. Obesity is associated with its downregulation, while LCN13 therapies in mice with either genetic or dietary obesity lead to an improvement in their hyperglycemia, hyperinsulinemia, insulin resistance, glucose intolerance, and hepatic steatosis [18, 19]. A recent study highlights the local and not systemic action of hepatic LCN13 in steatosis [20].

*Asns* is a GH-dependent male-predominant gene that encodes an enzyme involved in the synthesis of asparagine, an amino acid essential for cell growth and survival. It regulates plasma glucose levels [21], and its liver deletion reduces plasma glucose levels [22], while a high-fat diet upregulates its liver expression levels [23]. It is a potential biomarker associated with progression to liver fibrosis and HCC [24], and its hepatic expression was proposed as an independent predictor of overall survival in HCC [25].

HAO2 oxidizes several l-2-hydroxyacids. In HCC, a decrease in HAO2 may represent an early event in carcinogenesis in humans and rodents, and correlates with an unfavorable prognosis, while the reintroduction of *HAO2* in HCC cells decreases tumorigenesis [26, 27]. Of note, *HAO2* is a female-predominant gene, and HCC is more prevalent in men [28]. In nude mice, tumor overexpression of HAO2 inhibited cell proliferation, migration, and invasion [27]. Nevertheless, in acute liver failure, bioinformatic analysis of microarray datasets indicated that *HAO* was one of the 7 overexpressed genes [29].

*Pgc1a*, a GH-dependent female-predominant gene, is a transcriptional coactivator of genes that regulate mitochondrial biogenesis, respiration, and hepatic gluconeogenesis [30]. Interestingly, *Pgc1a^−/−^* mice, especially females, develop abnormally increased body fat, and following short-term starvation, hepatic steatosis [31]. Therefore, it has been suggested as a transcriptional target in hepatic steatosis [32]. Furthermore, in male rats, overexpression of liver *Pgc1a* reduced triacylglycerol [33], while hepatic disruption of *Pgc1a* evoked a 70% greater high-fat/high-sucrose diet-induced weight gain compared to wild-type mice [34]. This may indicate that *Pgc1a* levels that are higher in female compared to male livers may be protective against steatosis development in females.

*Hamp2* is a GH-dependent female-predominant gene, originally described for its antimicrobial activity [35], and it is known as a regulator of iron utilization [36]. In mice, 2 genes of this hepcidin are synthesized, *Hamp* and *Hamp2* [37]. Iron (Fe)-catalyzed oxidative damage is considered to be a causal factor in the development of hepatic fibrosis and HCC [38]. In this context, environmental toxicants, which play a role in the development of the metabolic syndrome and NAFLD repressed hepatic expression of hepcidin, resulting in an increase in serum Fe with accumulating Fe spilling into urine [39].

We compared the hepatic expression levels of *Lcn13*, *Asns*, *Hao2*, *Hamp2*, *and Pgc1a* in multiple mouse lines with altered GH production or action, focusing on sexual dimorphism in the search of druggable sex-related vulnerabilities in liver disease. The simultaneous analysis of mouse lines allowed evaluation of first, the comparison of the impact of different alterations of circulating GH/IGF1 levels in the context of normal hepatic GHR signaling, and second, the impact of liver *Ghr* ablation with or without *Stat5b* re expression. In the first case we included 4 transgenic mouse models: (i) central knockout for dopamine receptor D2 (NeuroDrd2KO) mice that have reduced GHRH leading to GH/IGF1-deficiency [40]; (ii) a somatotrope-specific knockout for both IGF1 (*Igf1r)* and insulin receptors (*Insr*), which leads to loss of negative feedback and elevation in circulating GH and IGF1 (HiGH mice) [41]; (iii) adult-onset hepatocyte-specific *Igf1* knockdown (aHepIGF1kd), which leads to increased circulating GH due to the loss of IGF1 negative feedback; and (iv) mice expressing a constitutively active form of *Stat5b* (STAT5bCA) exclusively in hepatocytes to mimic a female-specific pattern of STAT5b activation [10]. In the second case, to determine the direct impact of hepatocyte *Ghr* loss, we used a mouse line of adult-onset hepatocyte-specific knockdown of *Ghr* (aHepGHRkd), with reduced hepatic *Igf1* expression and circulating IGF1 levels, loss of negative feedback, and increased circulating GH levels [42]. In subsets of aHepGHRkd mice, a constitutively active form of *Stat5b* (STATCA) was expressed exclusively in the hepatocytes [10]. Mouse line characteristics are summarized in Table 1. In addition, we compared the changes in the expression levels of these metabolic genes with well-known GH-dependent sexual dimorphic cytochrome genes, that is, the male-predominant *Cyp7b1* and the female-predominant *Cyp2a4* and *Cyp2b9* genes.

**Table 1.** Summary of experimental models harboring genetic modifications of GH signaling

Mouse line	Strain	Altered gene	Target cell	Strategy	Sex-Age	Serum GH (transgenic/control)	Serum IGF1 (transgenic/control)	Liver *Igf1* mRNA (transgenic/control)	Liver *Socs2* mRNA (transgenic/control)	*Liver Ghr* mRNA (transgenic/control)	References
NeuroDrd2KO	*Drd2 ^loxP/loxP^*	*Drd2*	neurons	Nestinp-Cre	Male-5 mo	0.38*,*^a^*	0.45*	0.37*		1.04	[43]
					Female-5 mo	0.38*,*^a^*	0.52*	0.38*		1.17	
HiGH	*IgfIr and Insr ^loxP/loxP^*	*Insr,Igf1r*	somatotropes	rGHp-Cre	Male-4 mo	3.5–4.2*	1.25*	1.27*		0.88	[41]
					Female-4 mo	1.5–5.0*	1.20*	1.22*		1.24	
aHepIGF1kd*^b^*	*Igf1 ^loxP/loxP^*	*Igf1*	hepatocytes	AAV-TBGp-Cre	Male-5 mo	4.4*	0.13*	0.02*	1.7	1.04	NP
					Female-5 mo	4.7*	0.25*	0.05*	2.9*	0.96	NP
aHepSTAT5bCA	Wild-type	*Stat5bCA*	hepatocytes	AAV-TBGp-STAT5bCA	Male-3.5 mo	(low)	(high)	2.00*	8.5*	1.78*	[10]
					Female-3.5 mo	(low)	(high)				NP
aHepGHRkd*^b^*	*Ghr ^loxP/loxP^*	*Ghr*	hepatocytes	AAV-TBGp-Cre	Male-6 mo	18.1*	0.1*	0.05*	0.078*	0.004*	[42, 44, 45]
					Female-6 mo	1.85	0.23*	0.42*	0.30*	0.008*	
AHepGHRkd+ aHepSTAT5bCa*^b^*	*Ghr ^loxP/loxP^*	*Ghr/Stat5bCA*	hepatocytes	AAV-TBGp-Cre + AAV-TBGp-STAT5bCA	Male-6 mo	2.42	0.63*	0.65	2.40*	0.0048*	[37]
					Female-6 mo	0.9	0.72	0.92	2.01*	0.012*	

Between brackets means predicted not measured. Abbreviations: AAV-TBGp, adeno-associated viral vector using thyroxine-binding globulin promoter to drive transgene expression; CA, constitutively active; Cre, Cre recombinase; NP, not published.

^*a*^GH concentration, ng/ug protein.

^*b*^After 7 days of AAV8-TBGp-Cre injection, the rest are 66 days after AAV8-TBG-pCre injection.

^*^ *P* ≤ .05 vs respective control.

## Materials and Methods

Mice were housed with lights on at 06:00 and off at 18:00 hours, at 22 to 24 °C, and maintained on a standard rodent chow diet. Female and male mice were 4 to 6 months of age, ad libitum fed animals were euthanized between 09:00 and 12:00 hours. These studies were approved by the Institutional Animal Care and Use Committees of the Jesse Brown VA Medical Center and University of Illinois at Chicago, and Instituto de Biología y Medicina Experimental, Buenos Aires (granted approval #07/2016; in accordance with the Division of Animal Welfare NIH, for the Institute of Biology and Experimental Medicine A#5072-01).

### Experimental Models

#### Mice lacking dopamine receptor D2 in neurons: NeuroDrd2KO

To ablate dopamine D2 receptors from cells of neural origin, *Drd2^loxP/loxP^* mice [46] were crossed with B6.Cg-*Tg(Nes*-*cre)*  ^1Kln/J^ to obtain cohorts of *Drd2^loxP/loxP^* (control mice) and *Drd2^loxP/loxP^*.B6.*Cg*-*Tg(Nes*-*cre)*  ^1Kln/J^ littermates [40]. Thereafter, breeding pairs of *Drd2^loxP/loxP^* and *Drd2^loxP/loxP^,Tg(Nes*-*Cre)*  ^1Kln/J^ mice were used to generate *Drd2^loxP/loxP^* (control) and *Drd2^loxP/loxP^,Tg(Nes-Cre)* (NeuroDrd2KO) littermates [40]. Body weight was lower in male and female NeuroDrd2KO mice compared with sex-matched *Drd2 ^loxP/loxP^* mice. They had a decreased GH axis with lower GH pituitary content, *Igf1* liver mRNA, and serum IGF1 (Table 1) [43]. These alterations may be accounted for *Drd2* ablation from neurons, though it has also been suggested that brain expression of hGH may be involved [47].

#### Mice with elevated GH/IGF1 model: HiGH

This model was obtained by somatotrope-specific Cre-mediated inactivation of IGF1 receptor (*IgfIr*) and insulin receptor (*Insr*) genes [41]. *IgfIr,Insr ^loxP/loxP^* were used as controls. HiGH mice have elevated GH and IGF1 (Table 1) and a modest increment in body weight. Female HiGH mice are protected from some of the deleterious effects of high-fat feeding [41].

#### Mice without hepatic *Igf1* expression: aHepIGF1kd

The aHepIGF1kd mice were obtained by injecting *Igf^loxP/loxP^* mice (Jax Mice #016831**)** in the lateral vein 1.5 × 10^11^ genome copies of adeno-associated virus (AAV) bearing hepatocyte-specific thyroxine-binding protein (TBGp) promoter driving a Cre recombinase transgene (AAV8-TBGp-Cre; Cat #107787-AAV8, AAV.TBG.PI.Cre.rBG [AAV8], Addgene, Watertown, MA, diluted in 100 μL sterile phosphate-buffered saline [PBS]), or a null allele (AAV8-TBGp-Null vector [Addgene]), referred to as Null, or controls. Sixty-six days later animals were euthanized and the livers were collected. These mice have high GH levels due to the loss of IGF1 negative feedback (Table 1).

#### Mice with constitutive hepatic *Stat5b* expression: STAT5bCA

Constitutively active *Stat5b* hepatocyte expression was obtained by injecting in the lateral vein 10- to 12-week-old female and male mice with 0.75 × 10^11^ GC of AAV8 bearing the *Stat5b* gene (AAV-TBGp-STAT5b CA, 0.75 × 10^11^ GC per mouse), diluted in sterile PBS, with the dose of AAV8-Null vector adjusted to equalize the total GC of AAV8 per mouse across groups [10]. Sixty-six days later animals were euthanized and the livers were collected. Described in [10].

#### Adult-onset hepatocyte-specific GHR knockdown: aHepGHRkd and STAT5bCA reconstitution

To generate aHepGHRkd and STAT5bCA in aHepGHRkd mice, 10- to 12-week-old *Ghr ^loxP/loxP^* mice were injected in the lateral tail vein with 1.5 × 10^11^ GC of AAV bearing hepatocyte-specific TBGp promoter driving a Cre recombinase transgene (AAV8-TBGp-Cre; Cat #107787-AAV8, AAV.TBG.PI.Cre.rBG [AAV8], Addgene, Watertown, MA, diluted in 100 μL sterile PBS), or a null allele (AAV8-TBGp-Null vector [Addgene]), referred to here as Null, and used as a control. Expression of Cre recombinase exclusively in hepatocytes, leads to recombination of the floxed *Ghr* allele and knockdown of the hepatic *Ghr* mRNA and protein [42]. The generation of this model has been previously described [42, 44, 48]. Sixty-six days later, animals were euthanized and the livers were collected. In contrast to congenital models of *Ghr* ablation, in aHepGHRkd mice hepatic GH resistance occurs after sexual maturation. Livers of male aHepGHRkd mice exhibit slow onset steatosis associated with increased de novo lipogenesis, and modest hepatocyte ballooning and inflammation, while female aHepGHRkd are relatively protected against steatosis and liver injury [42, 44, 45]. To reconstitute hepatocyte STAT5b expression in aHepGHRkd, a subset of mice were co-injected with AAV8-TBGp-mStat5bCA (0.30 or 0.75 × 10^11^ GC per mouse); the STAT5bCA construct was validated and described in [10].

### Tissue RNA Extraction, Total cDNA Preparation, and Quantitative Real-Time PCR

After euthanasia, liver samples were frozen; thereafter, approximately 50 mg of the tissue was homogenized and RNA extracted, and then the first-strand cDNA was synthesized from 1 µg of total RNA as described [43].

Sense and antisense oligonucleotide primers (Invitrogen, Argentina) were designed based on published results or by the use of PrimerBlast (http://www.ncbi.nlm.nih.gov/tools/primer-blast/). The sequences are described in Table 2.

**Table 2.** Primers used in mRNA studies

Gene	Strand	Primer sequence (5′-3′)	Source
*Asns*	*Sense*	*TCCAACCGGTCTTGTCACT*	Designed by PrimerBlast
	*Antisense*	*ATCGCACTCAGACACTGCAC*	
*Lcn13*	*Sense*	*ACGTCGTCATTCGGGATGG*	Designed by PrimerBlast
	*Antisense*	*GAGAGGTGTGGTGAAGGGATG*	
*Cyp2b9*	*Sense*	*CTGAGACCACAAGCGCCAC*	Designed by PrimerBlast
	*Antisense*	*CTTGAGCATGAGCAGGACTCC*	
*Cyp7b1*	*Sense*	*TGAGGTTCTGAGGCTGTGCTC*	[43]
	*Antisense*	*TCCTGCACTTCTCGGATGATG*	
*Cyp2a4*	*Sense*	*AGCAGGCTACCT TCGACTGG*	[43]
	*Antisense*	*GCTGCTGAAGGCTATGCCAT*	
*Hao2*	*Sense*	*GCTTTGCATGGCTACCTCTAC*	Designed by PrimerBlast
	*Antisense*	*ATTGCTGGGCCGGTAGTTTC*	
*Hamp2*	*Sense*	*ACAGCCACCACACAAGTCC*	Designed by PrimerBlast
	*Antisense*	*CAATGTCTGCCCTGCTTTCT*	
*Pgc1a*	*Sense*	*TCTCAGTAAGGGGCTGGTTG*	Designed by PrimerBlast
	*Antisense*	*TTCCGATTGGTCGCTACACC*	
*Cyclophilin*	*Sense*	*CAGAACAT ATCCCTGCAT*	Designed by PrimerBlast
	*Antisense*	*GTTCAGCTCTGGGATGACCTT*	

Quantitative measurements of specific mRNA levels were performed by kinetic PCR as described [43]. Each sample was analyzed in duplicate and cyclophilin was used as housekeeping gene, and its cycle thresholds (CTs) did not show sex differences and were not altered by genotype.

### Statistical Analysis

Results are expressed as means ± SEM. The differences between means were analyzed by Student *t* test (only 2 groups); and 2-way ANOVA for independent measures for the effects of sex and genotype followed by Newman-Keuls test or Tukey honest significant difference test for unequal N. If *P* of the interaction was significant, individual means were compared by Tukey honest significant difference test; if not significant, groups of means (main effects) were analyzed by the same test. Kruskall-Wallis test was performed when a non-parametric test was necessary. *P* ≤ .05 was considered significant.

### Liver RNAseq Differential Expression Analysis

RNAseq count data obtained from male and female livers (6 mice/sex) from GHR-intact controls, and aHepGHRkd mice (available in the Gene Expression Omnibus [GEO] repository, accession number GSE154217) were reanalyzed in R. These samples were taken 8 months post-AAV injection performed between 10 and 12 weeks of age [45]. The multidimensional scaling plot as well as boxplots of the transformed counts to test homogeneity were done for aHepGHRkd and Null males, aHepGHRkd and Null females with the edgeR package, followed by the limma package with its *voom* method, to assess differential expression. The resulting *P* values were adjusted using the Benjamini and Hochberg method to control the False Discovery Rate. Genes with an adjusted *P* value <.05 were considered as differentially expressed.

Pathway enrichment analyses Gene Ontology (GO) for differentially expressed genes (DEGs) with > 1.5-fold change and adjusted *P* ≤ .05 were implemented using the clusterProfiler package.

## Results

### Male-Predominant Genes Are Generally Altered in Male and Not Female Livers, in Mouse Models With Low or Constant GH Levels in the Context of Preserved Hepatic GHR

**Figure 1.**
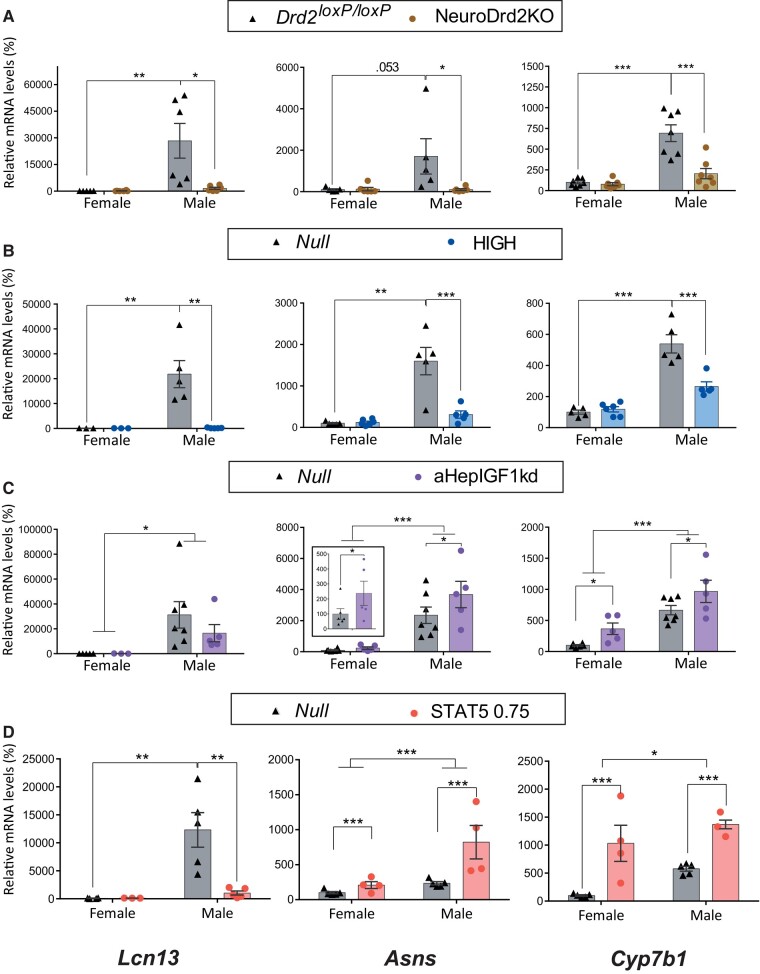
Male-predominant *Lcn13*, *Asns*, *and Cyp7b1* mRNA expression in mouse models with changes in circulating GH/IGF1 levels, in the context of normal hepatic GHR signaling. *Lcn13*, *Asns*, *and Cyp7b1* mRNA levels in female and male livers from A) NeuroDrd2KO and their control *Drd2^loxp/loxP^* mice (N between 5 and 7; male *Drd2^loxP/loxP^* vs male NeuroDrd2KO, *P* = .0066, *P* = .041, and *P* = .0002 for *Lcn13*, *Asns*, *and Cyp7b1*, respectively), B) HiGH and their control Null mice (N = between 5 and 6, except for *Lcn13* in females N = 3; male Null vs male HiGH *P* = .002, *P* = .00002, and *P* = .00031 for *Lcn13*, *Asns*, *and Cyp7b1*, respectively); C) aHepIGF1kd and their control Null mice (N between 5 and 7, except for *Lcn13* in females N = 5 and 3, main effect of genotype *P* = .039 and *P* = .0099 for *Asns* and *Cyp7b1*, respectively, and main effect of sex (upper bars) = .016, < .0001, and *P* = .0001 for *Lcn13*, *Asns*, *and Cyp7b1*); and D) Mice with constitutive expression of STAT5b (STATCA 0.75, 0.75 × 10^11^ GC per mouse; N = between 5 and 4, except for *Lcn13* in females N = 4, 3; main effect of genotype *P* = .0005 and *P* ≤ .0001 for *Asns* and *Cyp7b1*, respectively, and male Null vs male STAT5bCA 0.75 *P* = .0025 for *Lcn13*). Percentage of target mRNA levels (detailed at the bottom of the column) normalized to cyclophilin mRNA levels, in relation to control females (100%) are represented in the Y axis, * ≤ *P* .05, ** *P* ≤ .005, *** *P* ≤ .0005. When *P* values close to .05 were observed, the exact *P* value was included.

*Lcn13*, *Asns*, *and Cyp7b* mRNA expression levels were on average 267-, 18-, and 5-fold higher in control males compared to control females across models tested (Fig. 1).

Low GHRH/GH levels in the NeuroDrd2KO, as well as constant mildly high GH levels in HiGH mice lowered the 3 male-predominant genes in males, and abolished sex differences, while no changes in these male-predominant genes were observed in female livers (Fig. 1A and 1B).

Interestingly, disruption of liver *Igf1* expression which leads to GH increase (aHepIGF1kd mice), and constitutive hepatocyte *Stat5b* expression (STAT5bCA mice) which mimics a female-specific pattern of STAT5b activation, upregulated *Asns* and *Cyp7b1* in both sexes (Fig. 1C and 1D), preserving sex differences. In contrast, the male-predominant gene *Lcn13* was downregulated in male STAT5bCA livers (Fig. 1D).

### Female-Predominant Genes Are Altered in a Gene-Specific Manner in Mouse Models With Low or Constant GH Levels, in the Context of Preserved Hepatic GHR

**Figure 2.**
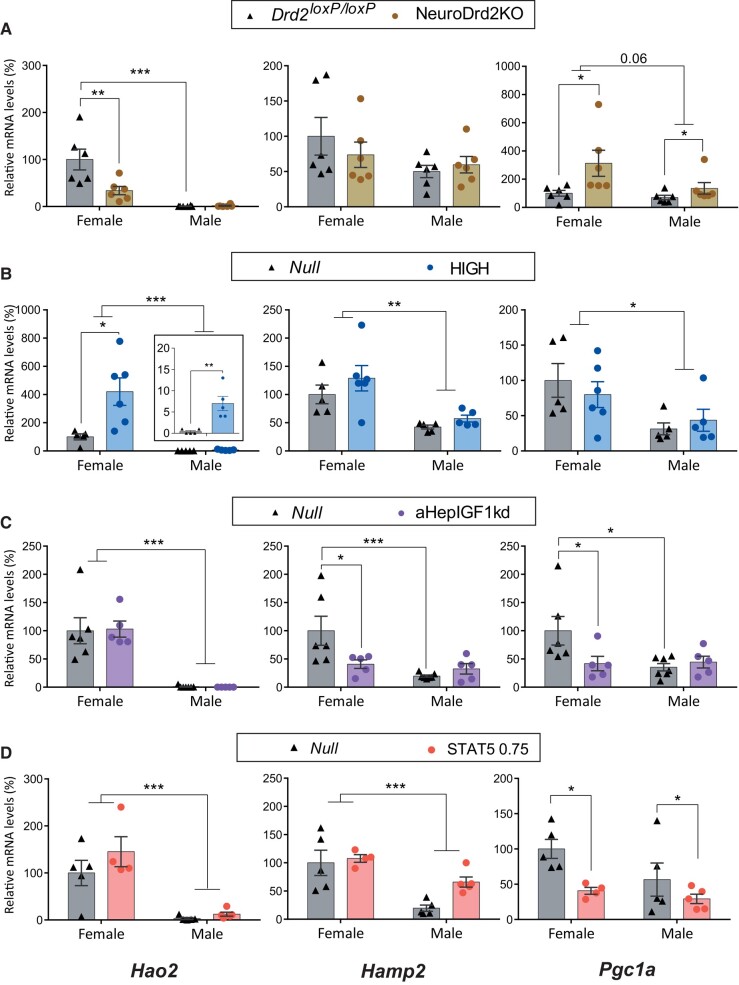
Female-predominant *Hao2*, *Hamp2*, *and Pgc1a* mRNA expression in mouse models with changes in circulating GH/IGF1 levels, in the context of normal hepatic GHR signaling. *Hao2*, *Hamp2*, *and Pgc1a* mRNA levels in female and male livers from A) NeuroDrd2KO and their control *Drd2^loxp/loxP^* mice (N = 6; *Pgc1a*: main effect of genotype *P* = .06; female *Drd2^loxP/loxP^* vs female NeuroDrd2KO: *P* = .0045 for *Hao2;* for *Pgc1a* main effect of sex *P =* .06); B) HiGH and their control Null mice (N = between 5 and 6; male *Null* vs male HiGH *P* ≤ .0001 for *Hao2*; female *Null* vs female HiGH: *P* = .0086 for *Hao*2, and main effect of sex *P =* .0003, *P* = .0007 and *P* = .0086 for *Hao2*, *Hamp2*, and *Pgc1a*, respectively, upper bars); C) aHepIGF1kd and their control Null mice (N between 5 and 7; female *Null* vs female aHepIGF1kd *P* = .050 and *P* = .018, for *Hamp2* and *Pgc1a*, respectively; and main effect of sex *P* < .0001 for *Hao2*); D) Mice with constitutive expression of STAT5b (STATCA 0.75, 0.75 × 10^11^ GC per mouse; N = between 5 and 4; main effect of genotype *P* = .011 for *Pgc1a*, and main effect of sex *P* < .0001 and *P* = .0004 for *Hao2* and *Hamp2*, respectively). For all panels, in the Y axis the percentages of target mRNA levels normalized to cyclophilin mRNA levels in relation to control females (100%) are represented (gene detailed at the bottom of the column). * ≤ *P* .05, ** *P* ≤ .005, *** *P* ≤ .0005. When *P* values close to .05 were observed, the exact *P* value was included.

**Figure 3.**
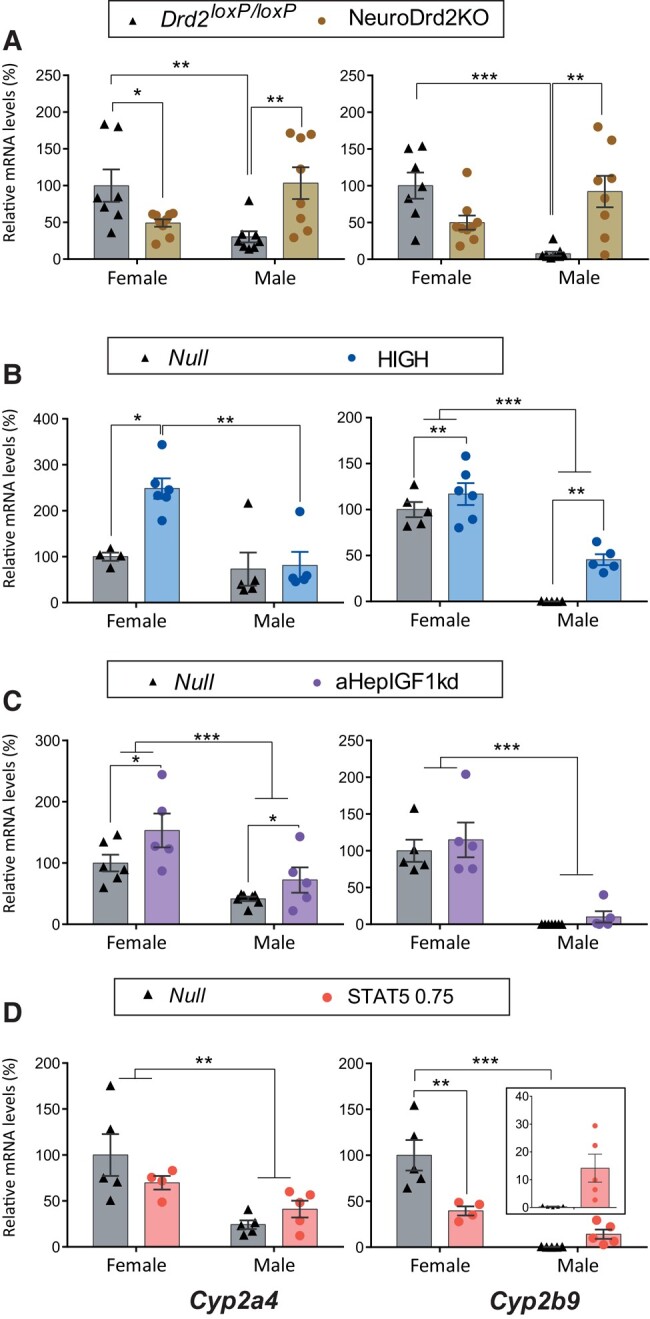
Female-predominant *Cyp2a4* and *Cyp2b9* mRNA expression in mouse models with changes in circulating GH/IGF1 levels, in the context of normal hepatic GHR signaling. *Cyp2a4 and Cyp2b9* mRNA levels in female and male livers from A) NeuroDrd2KO and their control *Drd2^loxp/loxP^* mice (N between 6 and 9; female *Drd2^loxP/loxP^* vs female NeuroDrd2KO: *P* = .026 for *Cyp2a4*; male *Drd2^loxP/loxP^* vs male NeuroDrd2KO *P* = .002 and *P* = .0008 for *Cyp2a4* and *Cyp2b9*, respectively); B) HiGH and their control Null mice (N = between 4 and 6; female *Null* vs female HiGH: *P* = .0078 for *Cyp2a4;* male *Null* vs male HiGH main effect of genotype *Cyp2b9*: *P* = .0019, and main effect of sex *P* < .0001 for *Cyp2b9*); C) aHepIGF1kd and their control Null mice (N between 5 and 7; main effect of genotype *P* = .021 for *Cyp2a4;* and main effect of sex *P* = .0005 and *P* < .0001 for *Cyp2a4* and *Cyp2b9*, respectively); D) Mice with constitutive expression of STAT5b (STATCA 0.75, 0.75 × 10^11^ GC per mouse; N = between 5 and 4; female Null vs female STAT5bCA 0.7 *P* = .0026 for *Cyp2b9*, and main effect of sex *P =* .0016 for *Cyp2a4*). For all panels, in the Y axis the percentages of target mRNA levels normalized to cyclophilin mRNA levels in relation to control females (100%) are represented (gene detailed at the bottom of the column). * ≤ *P* .05, ** *P* ≤ .005, *** *P* ≤ .0005.

The female-predominant genes *Hao2*, *Hamp2*, *Pgc1a*, *Cyp2a4*, *and Cyp2b9 were* on average 27-, 3.1-, 2.7-, 2.7-, and 530-fold higher in control females compared to control males, respectively, across models tested (Figs. 2 and 3).

*In males*, low GH levels (NeuroDrd2KO) upregulated the female-predominant genes *Pgc1a*, *Cyp2a4*, and *Cyp2b9* (Fig. 2A and 3A), constant mildly high GH levels (HiGH) increased *Hao2* and *Cyp2b9* expression levels (Figs. 2B and 3B) while disruption of liver *Igf1* (aHepIGF1kd) increased *Cyp2a4* (Fig. 3C), and constitutive expression of *Stat5b* (STAT5bCA) marginally increased female-predominant genes *Hamp2* and *Cyp2b9* (not significantly in a two-way analysis of variance, but in a *t* test *Null* males vs STAT5, 0.75 Males: 0.0023 and 0.026, for *Hamp2 and Cyp2b9*, respectively; Figs. 2D and 3D) while *Pgc1a* was downregulated (Fig. 2D). Therefore, low or constant GH levels, or Stat5b action feminized some of the female-predominant genes in male livers.

*In females*, on the other hand, there was a differential susceptibility to GH changes according not only to the female-predominant gene but also to the mouse model. In NeuroDrd2KO female mice, *Hao2* and *Cyp2a4* levels decreased, while *Pgc1a* increased (Fig. 2A and 3A). In HiGH females, *Hao2*, *Cyp2a4*, *and Cyp2b9* increased (Fig. 2B and 3B) and in aHepIGF1kd females *Cyp2a4* levels increased (Fig. 3C), consistent with the feminizing action of constant GH levels. Unexpectedly, deletion of *Igf1* expression from hepatocytes (aHepIGF1kd) decreased *Hamp2* and *Pgc1a* in females (Fig. 2C). Therefore, even though aHepIGF1kd mice have high GH levels, male (*Asns* and *Cyp7b1*) and female-predominant (*Hamp2* and *Pgc1a*) did not have the expected female-specific response, suggesting a role for liver *Igf1* on gene expression independent of GH levels.

In addition, in female STAT5bCA livers, the female-predominant *Pgc1a* and *Cyp2b9* genes were downregulated (Fig. 2D and 3D), suggesting that constitutive activation of STAT5b expression may trigger different responses compared with physiological GH constant activation of pSTAT5b.

Therefore, in males, low or constant levels of circulating GH downregulate male-predominant genes, and upregulate most, though not all, female-predominant genes—with the interesting exception of *Asns* and *Cyp7b1* genes, which are upregulated in aHepIGF1kd and STAT5bCA males. In females, male-predominant genes are not modified, once again with the exception of *Asns* and *Cyp7b1*, which are upregulated in aHepIGF1kd and STAT5bCA females. On the other hand, female-predominant genes are differentially regulated, changes being gene-specific, and varying in the different mouse models.

### Male-Predominant and Female-Predominant Genes Are Altered in Both Sexes in Mouse Models With Adult-Onset Hepatocyte Knockdown of the GHR (aHepGHRkd)

**Figure 4.**
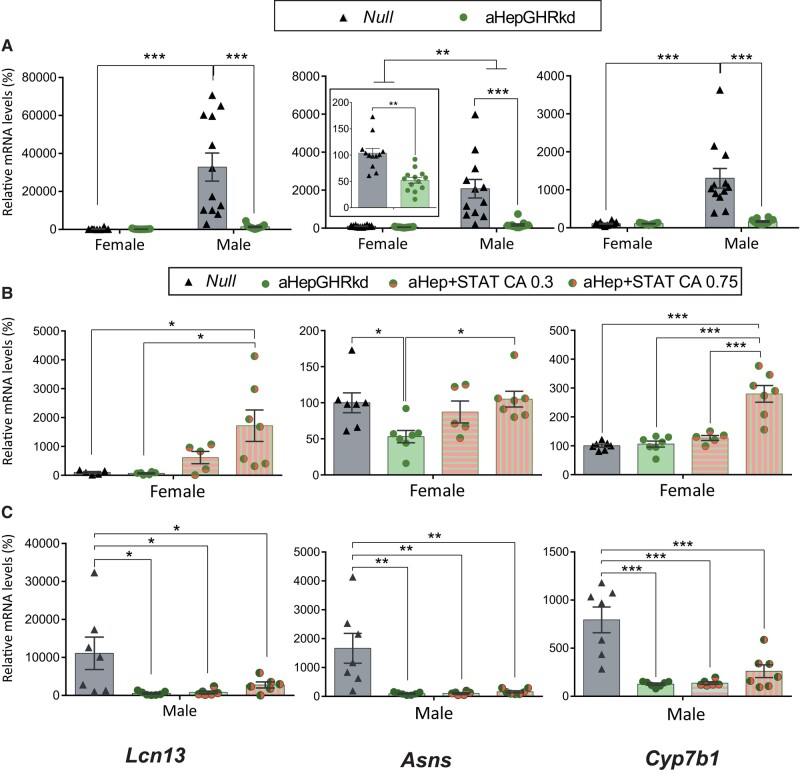
Male-predominant *Lcn13*, *Asns*, *and Cyp7b1* mRNA expression in mouse models with adult-onset hepatocyte knockdown of the GHR (aHepGHRkd), without or with reconstitution of STAT5b (aHepGHRkd + STATCA). *Lcn13*, *Asns*, *and Cyp7b1* mRNA levels in female and male livers from A) aHepGHRkd and their control Null mice (N = 10-13; male Null vs Male aHepGHRkd *P* = .00017, *P* = .00017 and *P* = .00018 for *Lcn13*, *Asns*, *and Cyp7b1*, respectively, and female Null vs female aHepGHRkd, *P* = .048 for *Asns;* and main effect of sex *P* = .004 for *Asns*, upper bar); B) Female Null, aHepGHRkd, and aHepGHRkd plus STATCA 0.35 or 0.75 × 10^11^ GC per mouse (N = 5-7, female aHepGHRkd vs female aHepGHRkd.STATCA 0.75: *P* = .011, *P* = .021, and *P* = .0001, for *Lcn13*, *Asns*, and *Cyp7b1*, respectively); C) male Null, aHepGHRkd, and aHepGHRkd plus STATCA 0.35 or 0.75 × 10^11^ GC per mouse (N = 6-7; *Lcn13*: *P* = .024, *P* = .012, and *P* = .03; *Asns*: *P* = .0020, *P* = .0044, and *P* = .0030; *Cyp7b1*: *P* = .00018, *P* = .0001, and *P* = .0003 for Null compared to aHepGHRkd, aHepGHRkd.STAT5bCA 0.35, and aHepGHRkd.STAT5bCA 0.75, respectively). For all panels, in the Y axis the percentages of target mRNA levels normalized to cyclophilin mRNA levels in relation to control females (100%) are represented (gene detailed at the bottom of the column). * ≤ *P* .05, ** *P* ≤ .005, *** *P* ≤ .0005.

**Figure 5.**
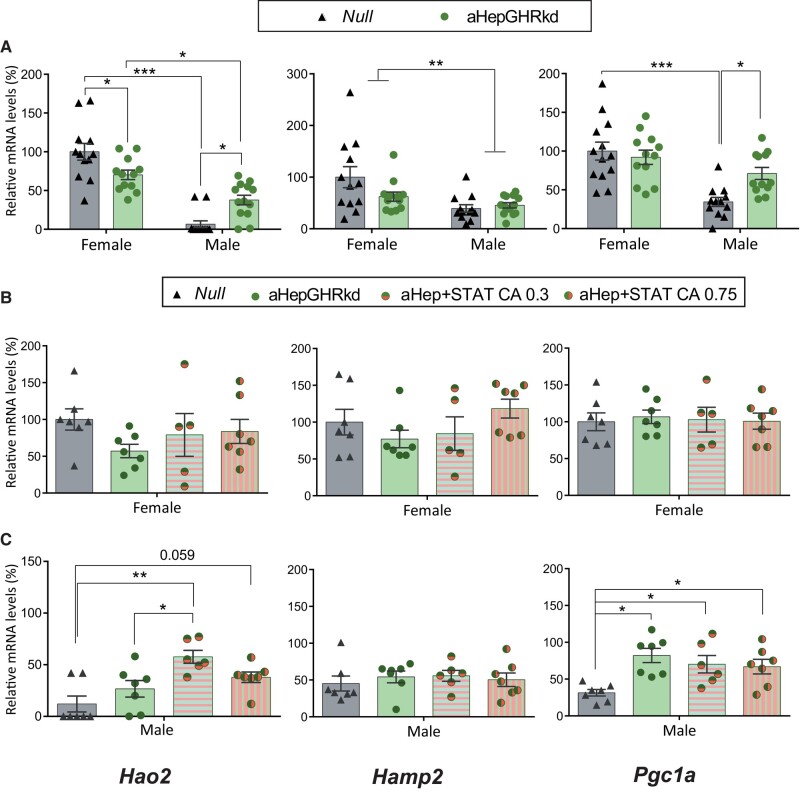
Female-predominant *Hao2*, *Hamp2*, *and Pgc1a* mRNA expression in mouse models with adult-onset hepatocyte knockdown of the GHR (aHepGHRkd), without or with reconstitution of STAT5b (aHepGHRkd + STATCA). *Hao2*, *Hamp2*, *and Pgc1a* mRNA levels in female and male livers from A) aHepGHRkd and their control Null mice (N = 12-13; male Null vs Male aHepGHRkd *P =* .015 and *P* = .03 for *Hao2* and *Pgc1a*, respectively, and female Null vs female aHepGHRkd *P =* .030 for *Hao2;* and main effect of sex *P* = .0019 for *Asns*); B) Female Null, aHepGHRkd, and aHepGHRkd plus STATCA 0.35 or 0.75 × 10^11^ GC per mouse (N = 5-7); C) male Null, aHepGHRkd, and aHepGHRkd plus STATCA 0.35 or 0.75 × 10^11^ GC per mouse (N = 6-7). For all panels, in the Y axis the percentages of target mRNA levels normalized to cyclophilin mRNA levels in relation to control females (100%) are represented (gene detailed at the bottom of the column). * ≤ *P* .05, ** *P* ≤ .005, *** *P* ≤ .0005.

**Figure 6.**
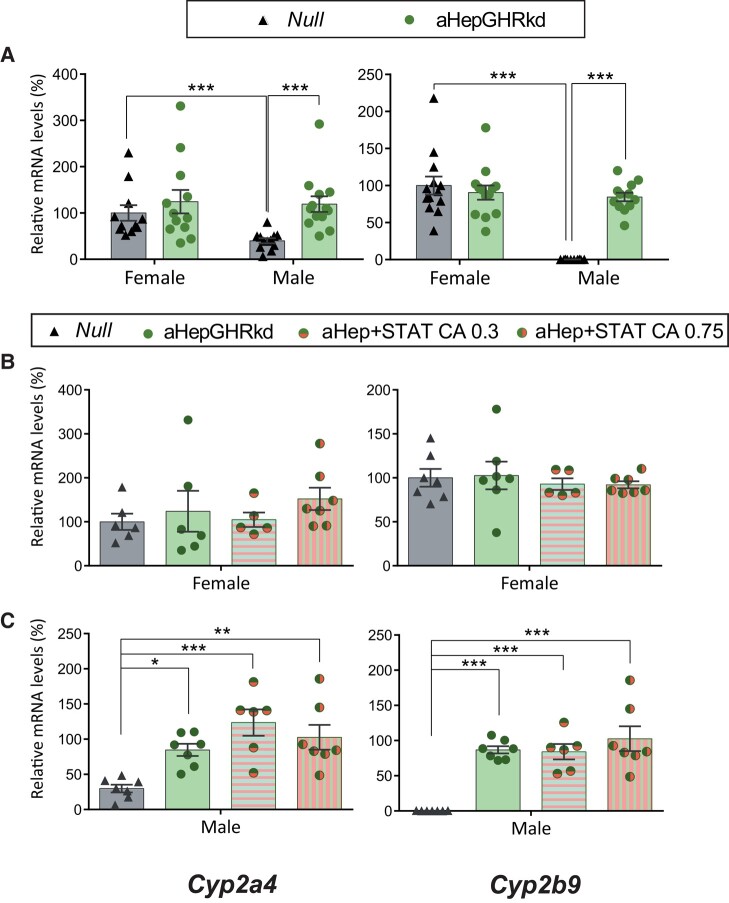
Female-predominant *Cyp2a4 and Cyp2b9* mRNA expression in mouse models with adult-onset hepatocyte knockdown of the GHR (aHepGHRkd), without or with reconstitution of STAT5b (aHepGHRkd + STATCA). *Cyp2a4 and Cyp2b9* mRNA levels in female and male livers from A) AHepGHRkd and their control Null mice (N between 10-13; male Null vs Male aHepGHRkd *P* ≤ .0001, and *P* = .0002 for *Cyp2a4* and *Cyp2b9*, respectively); B) Female Null, aHepGHRkd, and aHepGHRkd plus STATCA 0.35 or 0.75 × 10^11^ GC per mouse (N between 5 and 7); C) Male Null, aHepGHRkd, and aHepGHRkd plus STATCA 0.35 or 0.75 × 10^11^ GC per mouse (N between 6 and 7). For all panels, in the Y axis the percentages of target mRNA levels normalized to cyclophilin mRNA levels in relation to control females (100%) are represented (gene detailed at the bottom of the column). * ≤ *P* .05, ** *P* ≤ .005, *** *P* ≤ .0005.

Adult-onset *Ghr* knockdown from hepatocytes prevents GH action, and markedly lowered the 3 male-predominant genes (*Lcn13*, *Asns*, *and Cyp7b1)* in males and *Asns* in females (Fig. 4A*).* On the other hand, the female-predominant genes, *Hao2*, *Pgc1a*, *Cyp2a4*, and *Cyp2b9*, were upregulated in males (Figs. 5A and 6A) and unchanged in females, with the exception of decreased *Hao2* levels (Fig. 5A). For all genes, sexual dimorphism was lost (or decreased for *Asns and Hao2*), revealing the need of an intact *Ghr* for adequate differential expression of male and female-predominant genes in liver.

Therefore, loss of hepatic *Ghr* had a great impact on male-predominant genes that were downregulated, mostly resembling the outcome in NeuroDrd2KO mice with low serum GH levels. Nevertheless, in aHepGHRkd male mice, there was no increase in female-predominant genes as found in NeuroDrd2KO male mice. Disruption of liver *Ghr* had a lower impact in females compared to that in the NeuroDrd2KO mice.

### Effect of Constitutive Expression of STAT5b in aHepGHRKd Female and Male Mice

We studied whether STAT5b reconstitution in aHepGHRkd mice could revert the changes induced by loss of liver *Ghr* and the resulting lack of STAT5b activation. In female aHepGHRkd mice, *Stat5b* constitutive expression increased the 3 male-predominant genes compared to female aHepGHRkd mice (Fig. 4B), and *Lcn13* and *Cyp7b1* compared to Null mice. Therefore, the effect for these 2 genes seemed to be higher than just reconstitution of STAT5b in aHepGHRkd mice and was in concordance with the effect shown for STATCA expression in wild-type mice for the *Cyp7b1* gene (Fig. 1D).

On the other hand, in male aHepGHRkd mice, *Stat5b* constitutive expression did not modify the male-predominant genes, which remained low compared to Null male mice (Fig. 4C).

Female-predominant genes, on the other hand, were unmodified in aHepGHRkd female mice when *Stat5b* was constitutively expressed (Figs. 5B and 6B). Furthermore, in males, the increase in *Pgc1a*, *Cyp2a4*, and *Cyp2b9* in response to *Ghr* loss (Figs. 5A and 6A) was not modified by simultaneous expression of STAT5b (Figs. 5C and 6C), probably pointing to a prevalent effect of *Ghr* disruption. Therefore, even though STAT5b overexpression had effects in wild-type mice (Figs. 1D, 2D, and 3D), these effects were dampened by the loss of *Ghr*.

### Differential Response to the Loss of Hepatic *Ghr* in Liver Gene Expression in Both Sexes, Reanalyzing RNAseq Databases

Finally, to interpret results obtained in our selected genes in the context of large-scale gene variations, we reanalyzed RNAseq data from GEO (GSE154217) from male and female Null and aHepGHRKd mice [45]. We imported, organized, filtered, and normalized the data, using the edgeR package, and performed linear modeling and empirical Bayes moderation to assess differential expression and perform gene set testing using the limma package with its *voom* method.

**Figure 7.**
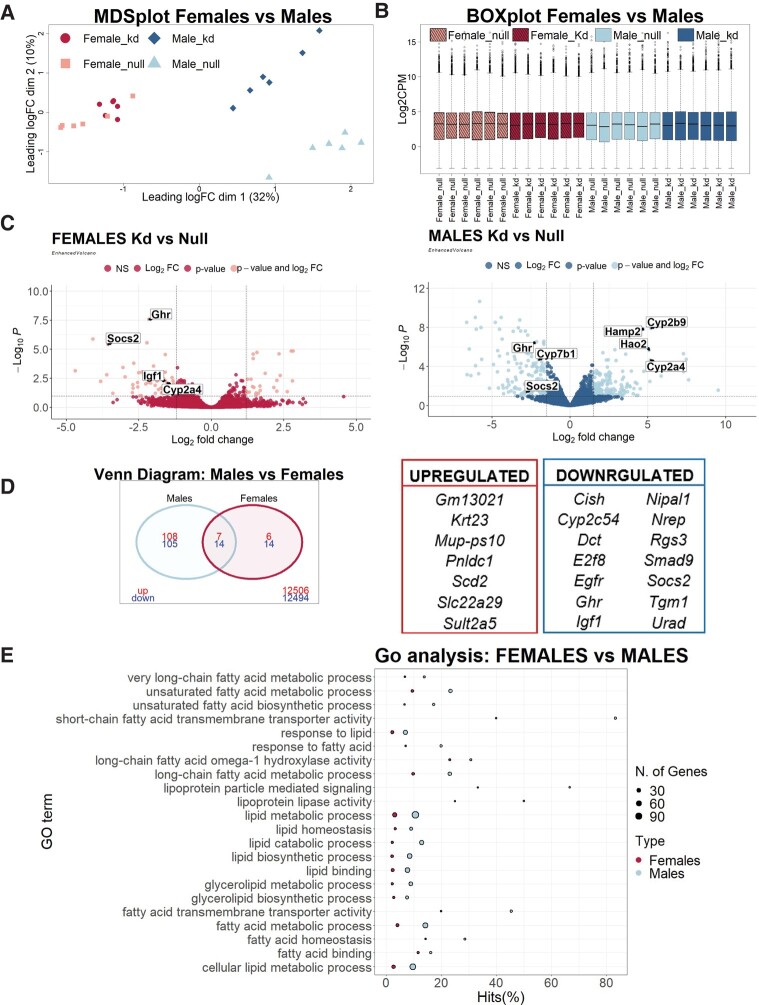
Liver transcriptome analysis of female and male Null and aHepGHRkd mice. Data re- analyzed from GEO data set GSE154217. A) MDS Plot of hepatic transcriptomes in control (Null), aHepGHRkd (kd) female and male mice. B) Boxplots of the transformed RNAseq counts across the 24 samples analyzed. C) Volcano Plots for females and males showing Log2 Fold Change (logFC) expression vs −Log10 (adj *P* Value) of differentially expressed genes (DEGs) (*adj *P* < .05; cutoff was set at 1.5). D) Venn diagram of the overlap between genes that were down- or upregulated in aHepGHRkd compared to Null in female or male mice. Number of genes in Venn diagrams vary from Volcano plots as the analysis considers only genes found in the datasets of both males and females. Right panel: list of up- and downregulated genes in both sexes. E) Plot showing Gene Ontology (GO) analysis results of significantly enriched GO terms filtered by *lipid*, *fatty acid*, or *sterol* of DEG in aHepGHRkd males and females (*P* value < .05). X axis (Hits %) equals the percentage of DEGs in relation to the number of genes associated with a GO term, while the size of the circle indicates the number of DEGs in each pathway.

MDS plot of principal components of the RNAseq data (Fig. 7A) generated separate clusters for sex on dimension 1, and within each sex the samples were separated into individual clusters for Null and aHepGHRKd based on dimension 2. Consistent with the modest impact of *Ghr* disruption on specific genes in female mice in the present work (see Figs. 4A, 5A, and 6A), MDS plot revealed an overlap in gene expression in females, in contrast with cluster separation in males according to genotype.

Boxplots of the transformed counts showed similar total counts across all 24 samples (Fig. 7B). Differential expression of genes (DEG: ≥ 1.5-fold difference, adjusted *P* < .05) identified 122 upregulated, 140 downregulated, and 12 821 unmodified genes in males, and 13 upregulated, 38 downregulated, and 13 352 unmodified genes in females (Supplementary Tables S1-S4) [49]. The comparison of Volcano plots (Fig. 7C) from males and females also revealed that fewer genes were differentially regulated in aHepGHRkd females (0.38%, compared to 2.00% in aHepGHRkd males). Within the Volcano plots, the sexually dimorphic genes analyzed in this study, as well as *Ghr*, *Igf1*, and *Socs2* are pointed. In the available data sets, *Lcn13* and *Asns* were not present. A Venn diagram showing the numbers of genes simultaneously altered by hepatic *Ghr* disruption in males and females (Fig. 7D) revealed a minimal overlap of DEG between sexes; only 5.8% of upregulated, and 10.5% of downregulated genes overlapped (Fig. 7D), highlighting the marked sex-specific gene response to loss of *Ghr*. The number of genes in Venn diagrams vary from Volcano plots, as the analysis considers only genes found in the datasets of both males and females. Of the overlapping downregulated genes, 29% were associated with GHR signaling (*Ghr*, *Igf*, *Socs2*, *Cish1*). Furthermore, of the overlapping downregulated genes, 4 were female-predominant (*Cish*, *Nipal1*, *Rgs3*, and *Tgm1)* and 2 were male-predominant (*Egfr* and *Ghr*), while of the upregulated genes, 3 were female-predominant (*Krt23*, *Mup-ps10*, and *Sult2a5*) and 1 was male-predominant (*Pnldc1*), indicating that 48% of the overlapping up- or downregulated genes were sexually dimorphic. These data were obtained from 12- to 13-month-old mice and cannot be strictly compared to real-time PCR analyses, which were performed in samples obtained from 6- to 7-month-old mice.

Finally, analysis of significantly regulated GO pathways comparing aHepGHRkd and GHR-intact livers from males and females revealed that more pathways were upregulated by *Ghr* loss in males compared with females (1349 and 803, in males and females, respectively) from a total of 21 333 and 21 403 pathways, respectively (Supplementary Tables S5 and S6) [50]. Notably fewer pathways were downregulated (310 and 99, in males and females, respectively). When regulated pathways were filtered for the terms *lipid*, *fatty acid*, or *sterol*, it became evident that lipid pathways were more upregulated in males compared to females (70 compared to 29; Supplementary Table S7) [51]. Of these pathways, 22 were significantly upregulated in the livers of both sexes, and the analysis of the comparative Hits (% of DEG of the pathway) as well as the number DEG (size of the circle) for each of these 22 pathways showed a greater upregulation of lipid pathways in males (Fig. 7E). In Supplementary Table S7 [51], the selected enriched GO terms filtered by lipid pathways, for aHepGHRkd vs Null males and females are detailed, and the 22 terms enriched in both sexes are highlighted in bold case.

## Discussion

Liver gene expression is sexually dimorphic depending mainly on GH. The complex and dynamic physiology of the liver discloses a clockwork precision in gene expression in order to cope with the metabolic needs particular to each sex. Although mostly a pulsatile pattern of GH secretion will enhance male-predominant genes and a constant female secretory GH pattern will increase female-predominant genes, a wide spectrum of responses has been detailed [10, 15]. Additionally, differential gene response varies according to zonation of the liver architecture [52] uncovering a sophisticated organ partly coordinated by brain neuroendocrine signals.

The maintenance of GH-dependent dimorphism is paramount for establishing a healthy liver metabolism as well as adequate drug and xenobiotic clearance, and its disruption has been associated with liver disease and sex-biased proneness to liver disorders [5]. Clinical and experimental data indicate that reductions in circulating GH levels or hepatic response are principal components of NAFLD. Importantly, it is well-known that humans have sex-biased proneness to different liver disorders [11-14, 53]. In particular, NAFLD is predominant in men compared to premenopausal women [11] and NAFLD occurrence may enhance the risk for liver fibrosis, and ultimately HCC. This different sex susceptibility is recapitulated in mouse models providing unique experimental tools to analyze gene responses to GH.

In the present work, using strategies aimed at altering different steps of the GH signaling process, we studied the sexually dimorphic response of several genes that participate in liver metabolism, and display a strong GH dependence. We used transgenic mouse lines in which growth hormone–releasing hormone (GHRH), liver GHR, STAT5b, or IGF1 were modified, leading to alterations in circulating GH and IGF1. Phenotypic sex differences have been described in some mouse models, although the underlying molecular causes remain to be elucidated. For example, in aHepGHRkd mice there is early hepatic de novo lipogenesis in both sexes, but only males develop steatosis [42]. Enhanced de novo lipogenesis and steatosis increase with age in males and are associated with hepatocyte ballooning, inflammation, and mild fibrosis [44]. Insulin resistance and increased triglycerides are also present in male aHepGHRkd mice, while systemic metabolic endpoints are only modestly modified in females, indicating that females are relatively protected from steatosis and liver injury that result from the loss of hepatic GHR. On the other hand, *Stat5b* deletion in the male mice liver leads to a 90% reduction of male-specific and 61% upregulation of female-specific genes, while in females more than 90% of sexually dimorphic genes remain unaltered [54-56]. In *Ghr*^−/−^ mice, increased adiposity and altered fasted glucose levels are more prominent in males than females [57]. These data point to a differential sex susceptibility in liver malfunction induced by altered GH signaling.

*Lcn13*, *Asns*, *Hao2*, *Hamp2*, *and Pgc1a* are GH-dependent sexually dimorphic liver genes which participate in liver glucose and/or lipid metabolism, and/or hepatocyte cellular proliferation. While *Cyp2a4*, *Cyp2b9*, and *Cyp7b1* encode cytochrome P450 enzymes, which catalyze oxidative conversion of steroids, lipids, and xenobiotics. Most of the work studying their physiological or pathological role was performed in one sex and the striking sex differences were not addressed. We therefore explored the different steps of the GH axis affecting the sex-dependent expression of these genes in 2 settings: (i) intact liver GHR expression but altered GH and IGF1 levels; and (ii) liver loss of GHR, with or without STAT5b reconstitution. Each mouse line is unique in its levels of serum GH, IGF1, and liver STAT5b or IGF1 expression, or GH resistance.

Our results demonstrate a specific regulation for each male- or female-predominant GH-dependent gene related not only to sex but also to the signaling pathway involved. Although gene alterations evoked by the different molecular approaches were more prominent in male livers, in concordance with the greater dependence on GH signaling, our results uncover unexpected gene responses in some mouse models which underline the complexity of liver metabolic regulation by GH.

*Lcn13* is a relatively low expressed gene, as revealed from RNAseq analysis, but with a striking sexual dimorphism—and interestingly in males, it was downregulated in every mouse model with the exception of aHepIGF1kd mice, indicating its great susceptibility to modifications of the GH signaling process. Of note, downregulation of its hepatic expression has been associated with obesity and altered glucose metabolism [18, 19].

*Asns* was decreased in males by low GH levels or action, or by constant GH levels, as expected for a male-predominant gene, but strikingly, the loss of liver *Igf1* or constant activation of STAT5b led to its upregulation in both sexes, suggesting that additional mechanisms participate in its regulation. A parallel behavior was observed for the *Cyp7b1* gene. Noteworthy, in RNAseq data recently published [10], *Cyp7b1* in males was not feminized by constant STAT5b activation and was even increased 2.3-fold, while constant GH infusion did feminize this gene (fold change −8.28). Of note, in that study, of a total of 176 male-biased genes, 62 were repressed in the male liver by constant GH infusion, but not by STAT5bCA, and 4 were unexpectedly induced by AAV8-STAT5bCA, similar to our findings for *Asns* and *Cyp7b1*, highlighting the heterogeneity of gene responses. Therefore, our results point to STAT5b-independent signaling pathways participating in GH action on *Asns* and *Cyp7b1* regulation [3, 58, 59]. Notably, in both STAT5bCA and aHepIGF1kd mice, suppressor of cytokine signaling 2 (*Socs2)* mRNA levels were increased, while it has been demonstrated that continuous GH administration does not increase *Socs2* [60]. From a metabolic context, ASNS is increased in the liver by NAFLD.

Higher levels of *Hamp2* and *Pgc1a* in females compared to males may be protective in terms of metabolic function. Even though sexual difference in *Pgc1a* expression was not particularly marked, higher levels in females were evidenced in most mouse models. Loss of liver *Igf1* and constant STAT5bCA led to *Pgc1a* decrease in females or in both sexes, respectively, which might be harmful in terms of metabolic liver health. In concordance, hepatic steatosis progressed in *Pgc1a^−/−^* mice, especially in females [61]. Furthermore, in patients with NAFLD, hepatic expression PGC1A was inversely correlated with liver fat and disease severity [62, 63]. Conversely, low GH levels in female and male NeuroDrd2KO mice, and disrupted GH action in male aHepGHRkd mice increased its expression levels, indicating GH may be inhibitory to this gene.

Expression levels of *Hamp2*, on the other hand, were largely unaffected in most mouse models. The only effect was observed in aHepIGF1kd females, in which decreased *Hamp2* expression was found. In this regard, it has been described that lowering *Hamp2* by environmental toxicants led to increased serum Fe, a causal factor in hepatic fibrosis [38, 39].

There are few data on the role of HAO2 in the liver besides its antiproliferative role in HCC cells [26], while its sexual dimorphism is noticeable. We demonstrate that *Hao2* expression levels decreased with low GH levels or action in females and increased with the constantly elevated GH levels in HiGH mice of both sexes, indicating the need for adequate GH levels, and the expected feminizing action of constant GH for this gene.

Even though pulsatile secretion could not be measured in all mouse models, major urinary proteins or MUPs [64] can be used an indirect parameter of GH pulsatility. These proteins are excreted in mouse urine, adult males secrete more than 3 times as much MUP as do females and importantly, their liver expression requires pulsatile occupancy of liver GH receptors [64]. A significant reduction of MUP production was published in NeuroDrd2KO males [40, 43], in aHepGHRkd male mice [45], and in STAT5bCA male mice [10], which may suggest reduced pulsatility in these groups. In concordance, in both NeuroDrd2KO and aHepGHRkd males, the 3 male-predominant genes were reduced, while the female-predominant genes *Cyp2a4*, *Cyp2b9*, and *Pgc1a* were upregulated. Nevertheless, in the STAT5bCA male mice, once again a differential response was found. Even though decreased pulsatility could be inferred from decreased MUP levels [10] only the male-predominant gene *Lcn13* was decreased, while unexpectedly, *Asns* and *Cyp7b1* mRNA levels were increased in both sexes. Furthermore, in aHepGHRkd females, the 3 male-predominant genes were increased by the higher dose of STAT5bCA, and in the case of *Cyp7b1* and *Lcn13*, expression levels increased also in comparison to Null mice. In this respect, physiological serum GH levels may generate different time-related signals or magnitude of STAT5b activation, compared with those reached in AAV8-STAT5bCA-infected hepatocytes, in which induced STAT5b levels may even exceed physiological levels of activated STAT5b. Moreover, GH also signals through other STAT-independent signal transduction pathways such as PI-3 kinase, ERK signaling, and Src family kinases [58, 59], which might not be activated in the STAT5bCA mouse model. Finally, though there are almost no IGF1Rs in the liver, except for stellate and Kupffer cells [65], a local action of liver IGF1 cannot be discarded, in view of the unexpected results found in the aHepIGF1kd model. Therefore, GH regulation of sexually dimorphic genes is far from linear, and although most male-predominant genes are downregulated by lack of pulsatility or constant GH infusion, there is a percentage which is not [10, 66]

Bioinformatic analyses helped to interpret results in the context of large-scale gene variations and were mostly in accordance with qPCR results in the aHepGHRkd mouse model. The few differences found (for example lack of *Hamp2* induction in aHepGHRkd mice by qPCR), may be related to the age of mice, or to the different analytical methods, as found when validating RNAseq data with qPCR. Results underscored the fact that GH modifies almost completely different gene signatures in each sex and highlighted the greater impact of *Ghr* loss in male compared to female mice for a wide variety of genes. Interestingly, annotation of these genes using the GO database showed that a large group of DEGs was associated with lipid metabolism, and differences were higher in males compared to females. This may underlie the greater susceptibility to steatosis of male compared to female aHepGHRkd mice and may be relevant in the study of therapies for liver disease.

There is almost no information on RNAseq data or gene expression levels in livers from patients with acromegaly or GH deficiency. In a recent randomized placebo-controlled trial in HIV patients, the GHRH analogue tesamorelin reduced liver fat and prevented fibrosis progression in HIV-associated NAFLD over 1 year [67]. In a transcriptomic analysis from paired liver biopsies specimens from this trial [68], *HAMP* mRNA was decreased after 1 year of tesamorelin vs placebo treatment, indicating a masculinization of the gene and in accordance with low *Hamp2* mRNA levels in female aHepIgf1kd, which have increased GH. Nevertheless, in the mentioned trial, there was no discrimination of results by sex.

On the other hand, in patients with acromegaly, gene expression signature has been described in adipose tissue [69], but the genes evaluated in our manuscript did not show significant changes, maybe pointing to organ-specific gene signatures in acromegaly.

Clinical trials have started to examine the impact of low-dose GH therapy to treat NAFLD (NCT02726542, NCT03375788) and [70]. It is therefore important to evaluate the impact of each therapy in a wide array of genes, which may modulate the ultimate clinical outcome. We demonstrate herein that important metabolic genes such as *Lcn13*, *Asns*, *Hao2*, *Hamp2*, *or Pgc1a*, are differentially modified by diverse strategies which alter the GH cascade. Our results showcase the complex and specific regulation of liver genes, which depends on not only the pattern or plasma levels of GH, and the presence of liver GHR, but also may be fine-tuned by the levels of STAT5b, other GH signaling pathway activation, and/or local *Igf1* synthesized. Hence, unraveling the intracellular pathways mediating the protective or harmful effects of specific liver genes should be considered when identifying the vulnerabilities and assets of GH therapeutic strategies.

## Data Availability

Some or all datasets generated during and/or analyzed during the current study are not publicly available but are available from the corresponding author on reasonable request.

## Current Affiliation for Andre Sarmento Cabral

Maimonides Institute for Biomedical Research of Córdoba (IMIBIC), Cordoba, Spain; Department of Cell Biology, Physiology, and Immunology, University of Córdoba, Cordoba, Spain; Hospital Universitario Reina Sofía (HURS), Cordoba, Spain; Centro de Investigación Biomédica en Red de Fisiopatología de la Obesidad y Nutrición, (CIBERobn), Cordoba, Spain.

## Abbreviations

AAV: adeno-associated virus
aHepGHRkd: adult-onset hepatocyte-specific *Ghr* knockdown
aHepIGF1kd: adult-onset hepatocyte-specific *Igf1* knockdown
*Asns*: asparagine synthetase
DEG: differentially expressed gene
GEO: Gene Expression Omnibus
GH: growth hormone
GHR: growth hormone receptor
GHRH: growth hormone–releasing hormone
*Hamp2*: hepcidin antimicrobial peptide 2
*Hao2*: hydroxyacid oxidase 2
HCC: hepatocellular carcinoma
HiGH: somatotrope-specific knockout for IGF1 and insulin receptors
IGF1: insulin-like growth factor 1
JAK2: Janus kinase 2
*Lcn13*: lipocalin 13
MUP: major urinary protein
NAFLD: nonalcoholic fatty liver disease
NeuroDrd2KO: dopamine receptor D2 knockout
PBS: phosphate-buffered saline
Pgc1a: peroxisome proliferator-activated receptor-gamma coactivator 1 alpha
STAT5b: signal transducers and activators of transcription 5b
STAT5bCA: constitutive hepatic *Stat5b* expression
TBGp: thyroxine-binding protein
